# A Little Energy Goes a Long Way: Build an Energy-Efficient, Accurate Spiking Neural Network From Convolutional Neural Network

**DOI:** 10.3389/fnins.2022.759900

**Published:** 2022-05-26

**Authors:** Dengyu Wu, Xinping Yi, Xiaowei Huang

**Affiliations:** ^1^Department of Computer Science, University of Liverpool, Liverpool, United Kingdom; ^2^Department of Electrical Engineering and Electronics, University of Liverpool, Liverpool, United Kingdom

**Keywords:** spiking neural network (SNN), spiking network conversion, deep learning, deep neural networks (DNNs), event-driven neural network

## Abstract

This article conforms to a recent trend of developing an energy-efficient Spiking Neural Network (SNN), which takes advantage of the sophisticated training regime of Convolutional Neural Network (CNN) and converts a well-trained CNN to an SNN. We observe that the existing CNN-to-SNN conversion algorithms may keep a certain amount of residual current in the spiking neurons in SNN, and the residual current may cause significant accuracy loss when inference time is short. To deal with this, we propose a unified framework to equalize the output of the convolutional or dense layer in CNN and the accumulated current in SNN, and maximally align the spiking rate of a neuron with its corresponding charge. This framework enables us to design a novel explicit current control (ECC) method for the CNN-to-SNN conversion which considers multiple objectives at the same time during the conversion, including accuracy, latency, and energy efficiency. We conduct an extensive set of experiments on different neural network architectures, e.g., VGG, ResNet, and DenseNet, to evaluate the resulting SNNs. The benchmark datasets include not only the image datasets such as CIFAR-10/100 and ImageNet but also the Dynamic Vision Sensor (DVS) image datasets such as DVS-CIFAR-10. The experimental results show the superior performance of our ECC method over the state-of-the-art.

## 1. Introduction

Spiking neural networks (SNNs) are more energy efficient than convolutional neural networks (CNNs) in inference time because they utilize matrix addition instead of multiplication. SNNs are supported by new computing paradigms and hardware. For example, SpiNNaker (Painkras et al., 2012), a neuromorphic computing platform based on SNNs, can run real-time billions of neurons to simulate the human brain. The neuromorphic chips, such as TrueNorth (Akopyan et al., 2015), Loihi (Davies et al., 2018), and Tianji (Pei et al., 2019), can directly implement SNNs with 10,000 neurons being integrated onto a single chip. Moreover, through the combination with sensors, SNNs can be applied to edge computing, robotics, and other fields, to build low-power intelligent systems (Pfeiffer and Pfeil, 2018).

However, the discrete nature of spikes makes the training of SNNs hard, due to the absence of gradients. This article follows a cutting-edge approach to obtaining a well-performed SNN by converting from a trained CNN of the same structure. This approach has an obvious benefit from the sophisticated training regime of CNNs, i.e., it is able to take advantage of the successful—and still fast improving—training methods on CNNs without extra efforts to adapt them to SNNs. Unfortunately, existing CNN-to-SNN conversion methods either cannot achieve a sufficiently small accuracy loss upon conversion (Rueckauer et al., 2017; Sengupta et al., 2019), or need a high latency (Sengupta et al., 2019), or require a significant increase in the energy consumption of the resulting SNNs (Han et al., 2020). Moreover, recent methods such as Han et al. (2020) do not work with the batch-normalization layer—a functional layer that plays a key role in the training of CNNs (Santurkar et al., 2018).

**Figure 1:**
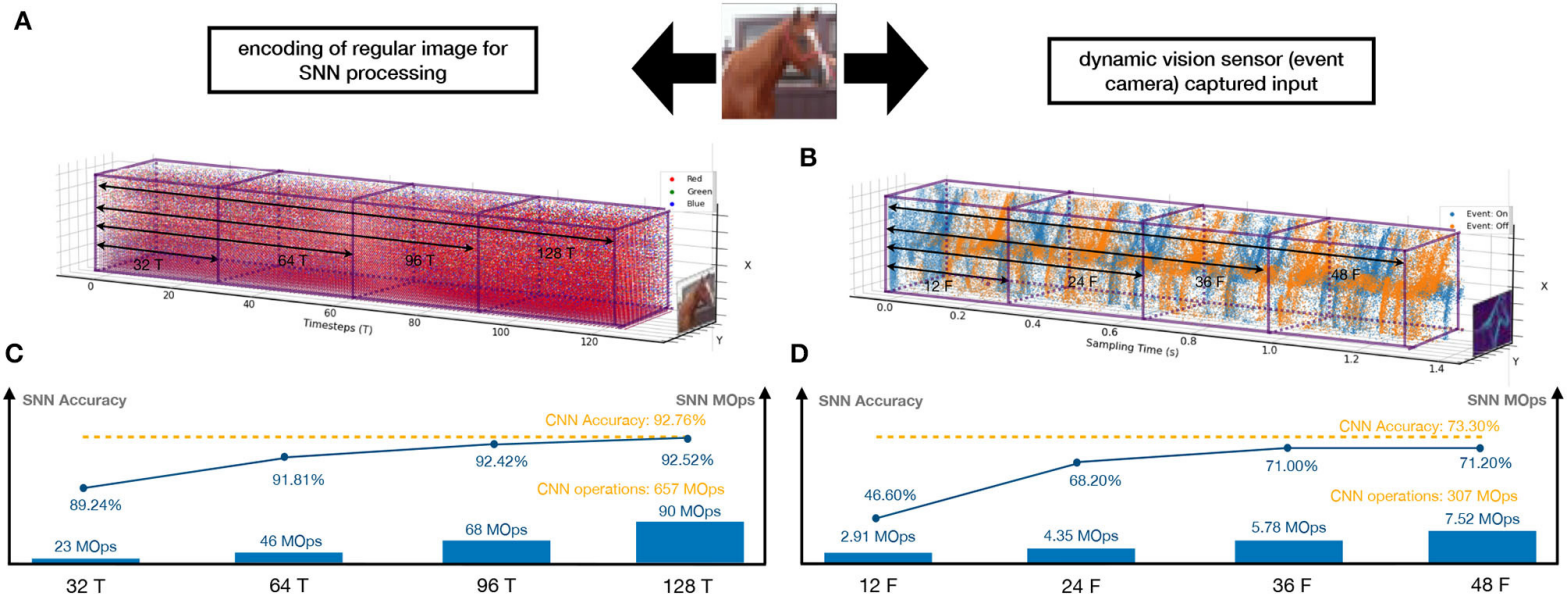
An illustrative diagram showing how SNNs process two different types of inputs and their performance comparison with CNNs. A regular image (left column)—taken from the camera—is preprocessed into a spike train **(A)**, which then runs through the SNN in several timesteps (e.g., 128 timesteps as in the figure). A DVS input—taken from the event camera—can be represented directly as a spike train **(B)**, and processed naturally by the SNN in several frames (e.g., 48 frames as in the figure). **(C,D)** show the SNN's performance with respect to the three objectives (accuracy, energy efficiency, and latency), compared to CNNs.

This article levels up the CNN-to-SNN conversion with the following contributions. First of all, methodologically, we argue that the conversion needs to be multi-objective—in addition to accuracy loss, energy efficiency and latency should be considered altogether. Figure 1 provides an illustration showing how SNNs process images and DVS inputs, exhibiting how well our methods enable the achievement of the three objectives and its comparison with CNNs. Actually, Figures 1C,D, SNNs can have competitive accuracy upon conversion (92.52 vs. 92.76% and 71.20 vs. 73.30%, respectively) and be significantly more energy efficient than CNNs (90 vs. 657 MOps and 7.52 vs. 307 MOps, respectively). While it is hard to compare the latency as SNNs and CNNs work on different settings, our method implements high energy efficiency with low latency (128 timesteps for images and 48 frames for DVS inputs). As shown in our experiments, ours are superior to the state-of-the-art conversions (Rueckauer et al., 2017; Sengupta et al., 2019; Han et al., 2020).

Second, we follow an intuitive view aiming to establish an equivalence between the activations in an original CNN and the current in the resulting SNN. This view inspires us to consider an explicit, and detailed, control of the current flowing through the SNN. Technically, we develop a unifying theoretical framework, which treats both weight normalization (Rueckauer et al., 2017) and threshold balancing (Sengupta et al., 2019) as special cases. Based on the framework, we develop a novel conversion method called explicit current control (ECC), which includes two techniques: current normalization (CN), to control the maximum number of spikes fed into the SNN, and thresholding for residual elimination (TRE), to reduce the residual membranes potential in the neurons.

Third, we include in ECC a dedicated technique called consistency maintenance for batch-normalization (CMB) to deal with the conversion of the batch-normalization layer.

Finally, we implement ECC into a tool SpKeras^1^ and conduct an extensive set of experiments on not only the regular image datasets, such as CIFAR-10/100 and ImageNet but also the Dynamic Vision Sensor (DVS) datasets such as DVS-CIFAR-10. Note that, DVS datasets are dedicated to SNN processing. The experimental results show that compared with state-of-the-art methods (Rueckauer et al., 2017; Sengupta et al., 2019; Han et al., 2020), ECC can optimize over three objectives at the same time, and have superior performance. Moreover, we notice that (1) ECC can utilize the conversion of batch-normalization to reduce the latency, and (2) ECC is robust to the hardware deployment because the quantisation—by using 7–10 bits to represent the originally 32-bit weights—does not lead to significant accuracy loss.

We remark that this article is not to argue for the replacement of CNNs with SNNs in general. Instead, we suggest a plausible deployment workflow, i.e., train a CNN → convert into an SNN → deploy on edge devices with e.g., an event camera. The workflow will not be a good option if any of the three objectives is not optimized.

## 2. Related Study

### 2.1. Current “Energy for Accuracy” Trend in CNN-to-SNN Conversion

A few different conversion methods, such as Diehl et al. (2015), Rueckauer et al. (2017), Sengupta et al. (2019), and Han et al. (2020), have been proposed in the past few years. It is not surprising that there is an accuracy loss between SNNs and CNNs. For example, in Rueckauer et al. (2017) and Sengupta et al. (2019), the gap is between 0.15 and 2% for CIFAR10 networks. A recent study by Sengupta et al. (2019) shows that this gap can be reduced if we use a sufficiently long (e.g., 1024 timesteps) spike train to encode an input. However, a longer spike train will inevitably lead to higher latency. This situation was believed to be eased by Han et al. (2020), which claim that the length of the spike train can be drastically shortened in order to achieve near-zero accuracy loss. However, as shown in Section 4.2 (**Figure 3A**), their threshold scaling method can easily lead to a significant increase in the spike-caused synaptic operations (Rueckauer et al., 2017), or spike operations for short, which also lead to a significant increase in the energy consumption.

Other related studies include (Rathi et al., 2020; Li et al., 2021; Rathi and Roy, 2021), which calibrate SNN to a specific timestep by gradient-based optimization. The calibration requires extra training time to find the optimal weights or hyper-parameters, such as some thresholds. In contrast, Deng and Gu (2021) trained a dedicated CNN for an SNN with fixed timesteps by shifting and clipping ReLU activations, although the accuracy loss of these SNNs cannot converge to zero when increasing the timesteps, as shown in **Figure 6B**. Besides, instead of reducing spikes, Lu and Sengupta (2020) explored SNN with binary weights to further improve the energy efficiency by consuming less memory.

### 2.2. Technical Ingredients in CNN-to-SNN Conversion

Table 1 provides an overview of the existing conversion methods (Cao et al., 2015; Diehl et al., 2015; Rueckauer et al., 2017; Sengupta et al., 2019; Han et al., 2020) and ours, from the aspects of technical ingredients and workable layers. In the beginning, most of the techniques, such as Cao et al. (2015) and Diehl et al. (2015), are based on hard reset (HR) spiking neurons, which are reset to fixed reset potential once their membrane potential exceeds the firing threshold. HR is still used in some recent methods such as Sengupta et al. (2019). The main criticism of HR is its significant information loss during the SNN inference. Soft reset (SR) neurons are shown better in other studies such as Rueckauer et al. (2017) and Han et al. (2020).

**Table 1:** Comparison of key technical ingredients (HR, SR, WN, TB, TS, ECC) and workable layers (BN, MP, AP) with the state-of-the-art methods.

	**HR**	**SR**	WN^*^	TB^*^	**TS**	**ECC**	BN^**^	**MP**	**AP**
Cao et al. (2015)	√								√
Diehl et al. (2015)	√		√						√
Rueckauer et al. (2017)		√	√				√	√	
Sengupta et al. (2019)	√			√					√
Han et al. (2020)		√		√	√				√
[This paper]		√	√	√		√	√		√

*HR, hard reset; SR, reset by subtraction, or soft reset; WN, weight normalization; TB, threshold balancing; TS, threshold scaling; ECC, explicit current control; BN, batch normalization; MP, max pooling; AP, average pooling*.

* *As a contribution to this article, in Section 3.2, we show that both WN and TB are special cases of our ECC framework*.

** *Among all methods, only those that can handle BN have bias terms in their pre-trained CNNs*.

Weight normalization (WN) is proposed in Diehl et al. (2015) and extended in Rueckauer et al. (2017) to regulate the spiking rate in order to reduce accuracy loss. The other technique, threshold balancing (TB), is proposed by Sengupta et al. (2019) and extended by Han et al. (2020), to assign appropriate thresholds to the spiking neurons to ensure that they operate in the linear (or almost linear) regime. We show in Section 3.2 that both WN and TB are special cases of our theoretical framework.

Another technique called threshold scaling (TS) is suggested by Han et al. (2020). However, as our experimental result is shown in **Figure 3A**, TS leads to significantly greater energy consumption (measured as MOps). On the other hand, our ECC method can achieve smaller accuracy loss and significantly less energy consumption.

We also note in Table 1 the differences in terms of workable layers in CNNs/SNNs for different methods. For example, the batch-normalization (BN) layer (Ioffe and Szegedy, 2015) is known as important for the optimization of CNNs (Santurkar et al., 2018), but only one existing method, i.e., Rueckauer et al. (2017), can work with it. Similarly, the bias values of neurons are pervasive for CNNs. Actually, the consideration of BN is argued in Sengupta et al. (2019) as the key reason for the higher accuracy loss in Rueckauer et al. (2017). The results of this article show that we can keep both BN and bias without significantly increased energy consumption, by maintaining the consistency between the behavior of SNN and CNN. BN can help with the reduction of latency. As we discussed earlier and in Section 5, our ECC method may be applicable to B-SNN and further improve its performance. Moreover, we follow most SNN research to consider the average pooling (AP) layer instead of the max pooling (MP) layer.

### 2.3. Direct Training

Spiking Neural Networks process information through non-differentiable spikes, and thus the backpropagation (BP) (LeCun et al., 1989) training algorithm cannot be directly applied. Few attempts by Lee et al. (2016) and Lee et al. (2020) have been made to adapt the BP algorithm by approximating its forward propagation phase. Such direct training requires high computational complexity to achieve an accuracy that is close to CNNs (Wu et al., 2021). Unlike these methods which approximate the BP algorithm (Lee et al., 2016, 2020), both of which may lead to performance degradation, we choose CNN-to-SNN conversion which can take full advantage of the continuously improving CNN training methods. Other than these methods which try to reproduce the success of CNN training, there are other direct training methods, such as approaches based on reservoir computing (Soures and Kudithipudi, 2019) and evolutionary algorithms (Schuman et al., 2020).

## 3. Explicit Current Control

By leveraging the correspondence between activation in CNNs and current in SNNs,^2^ we propose a unifying theoretical framework targeting multiple objectives, including accuracy, latency, and energy efficiency. Going beyond the existing conversion techniques (refer to Table 1) that consider some of the objectives individually, we view these multi-objective holistically through the lens of the unifying theoretical framework. Inspired by such a new viewpoint, we develop explicit current control (ECC) techniques to normalize, clip, and maintain the current through the SNNs for the purposes of reducing accuracy loss, latency, and energy consumption.

### 3.1. Existing CNN-to-SNN Conversion

Without loss of generality, we consider a CNN model of *N* layers such that layer *n* has *M*^*n*^ neurons, for *n*∈{1, 2, …, *N*}. The output of the neuron *i*∈{1, …, *M*^*n*^} at layer *n* with ReLU activation function is given by


(1) $$a_{i}^{n}=\max\left\{0,\sum_{j=1}^{M^{n-1}}W_{ij}^{n}a_{j}^{n-1}+b_{i}^{n}\right\}$$

where $W_{ij}^{n}$ is the weight between the neuron *j* at layer *n*−1 and the neuron *i* at layer *n*, $b_{i}^{n}$ is the bias of the neuron *i* at layer *n*, and $a_{i}^{0}$ is initialized as the input *x*_*i*_.

The activation $a_{i}^{n}$ indicates the contribution of the neuron to the CNN inference. For CNN-to-SNN conversion, the greater $a_{i}^{n}$ is, the higher the spiking rate will be, for the corresponding neuron on SNN. An explanation of a conversion method from CNNs to SNNs was first introduced by Rueckauer et al. (2017) by using data-based weight normalization.

The conversion method uses integrated-and-fire (IF) neurons to construct a rate-based SNN without leak and refractory time. If considering practical implementations, the rate-based SNN expects a relatively large interval between input spikes to minimize the effect of refractory time. To convert from a CNN, the spiking rate of each neuron in SNN is related to the activation of its corresponding neuron in the CNN. An iterative algorithm based on the *reset by subtraction* mechanism is described below. The membrane potential $V_{i}^{n}\left(t\right)$ of the neuron *i* at the layer *n* can be described as


(2) $$\begin{array}{c} V_{i}^{n}(t)=V_{i}^{n}(t-1)+Z_{i}^{n}(t)-\Theta_{i}^{n}(t)V_{thr}^{n} \end{array}$$

where $V_{thr}^{n}$ represents the threshold value of layer *n* and $Z_{i}^{n}\left(t\right)$ is the input current to neuron *i* at layer *n* such that


(3) $$Z_{i}^{n}(t)=\sum_{j=1}^{M^{n-1}}W_{ij}^{n}\Theta_{j}^{n-1}(t)+b_{i}^{n}$$

with $\Theta_{i}^{n}\left(t\right)$ being a step function defined as


(4) $$\Theta_{i}^{n}(t)=\{\begin{array}{cc} 1, & \text{if}V_{i}^{n}(t)\geq V_{thr}^{n}\\ 0, & \text{otherwise.} \end{array}$$

In particular, when the current $V_{i}^{n}\left(t\right)$ reaches the threshold $V_{thr}^{n}$, the neuron *i* at layer *n* will generate a spike, indicated by the step function $\Theta_{i}^{n}\left(t\right)$, and the membrane potential $V_{i}^{n}\left(t\right)$ will be reset immediately for the next timestep by subtracting the threshold.

### 3.2. A Unifying Theoretical Framework

The above CNN-to-SNN conversion method is designed specifically for weight normalization (Rueckauer et al., 2017), and cannot accommodate other conversion methods, e.g., threshold balancing (Sengupta et al., 2019). We propose a novel theoretical framework for CNN-to-SNN conversion that covers both weight normalization (Rueckauer et al., 2017) and threshold balancing (Sengupta et al., 2019) as special cases. In particular, the proposed framework improves over (Rueckauer et al., 2017) by adopting a thresholding mechanism to quantify the accumulated current into spikes in SNN and extends the threshold balancing mechanism to be compatible with batch normalization and bias.

We will work with the spiking rate of each SNN neuron *i* at layer *n*, defined as $r_{i}^{n}\left(t\right)=N_{i}^{n}\left(t\right)/t$, where $N_{i}^{n}\left(t\right)$ is the number of spikes generates in the first *t* timesteps by neuron *i* at layer *n*. We remark that it is possible that $r_{i}^{n}\left(t\right)>1$, i.e., multiple spikes in a single timestep, in which case the latency is increased to process extra spikes.

Our framework is underpinned by Proposition 1.

** Proposition 1**. In the CNN-to-SNN conversion, if the first layer CNN activation $a_{i}^{1}$ and the first layer SNN current $Z_{i}^{1}\left(t\right)$ satisfy the following condition


(5) $$\frac{1}{T}\sum_{t=1}^{T}Z_{i}^{1}(t)=a_{i}^{1},$$

where *T* is a predefined maximum timestep, then the SNN spiking rate at time step *t* can be iteratively computed by


(6) $$r_{i}^{n}(t)=\frac{1}{V_{thr}^{n}}\left(\overset{M^{n-1}}{\underset{j=1}{\sum^{\text{​}}}}W_{ij}^{n}r_{j}^{n-1}(t)+b_{i}^{n}\right)-\Delta_{i}^{n}(t)$$

with $\Delta_{i}^{n}\left(t\right)≜V_{i}^{n}\left(t\right)/\left(tV_{thr}^{n}\right)$ representing the residual spiking rate. Initially, the spiking rate of neuron *i* at the first layer is $r_{i}^{1}\left(t\right)=a_{i}^{1}/V_{thr}^{1}-\Delta_{i}^{1}\left(t\right)$.

** Remark 1**. The spiking rate in Equation (6) is a generalised form of those using weight normalization (WN) (Rueckauer et al., 2017) and threshold balancing (TB) (Sengupta et al., 2019). When keeping $V_{thr}^{1}=1$, by normalizing $W_{ij}^{n}$ we obtain WN; when keeping $W_{ij}^{n}$ unchanged, by normalizing $V_{thr}^{n}$ we obtain TB. When applying a scaling factor α^*n*^ to the threshold $V_{thr}^{n}$, Proposition 1 recovers (Han et al., 2020).

The condition in Equation (5) bridges between the activations in CNNs and the accumulated currents in SNNs, i.e., *within the duration of a spike train, the average accumulated current equals the CNN activation*. This is key to our theoretical framework, and different from some previous conversion methods such as Rueckauer et al. (2017), which bridges between activations and firing rates. This *activation-current association* is reasonable because it aligns with the intuitions that (i) given a fixed spiking rate, a greater CNN activation requires a greater accumulated current in the SNN; and (ii) given a pre-trained CNN, more input spikes lead to increased current in the SNN.

Proposition 1 suggests that an explicit, optimized control on the currents may bring benefits to the spiking rate (so as to reduce energy consumption) and the residual current (so as to reduce the accuracy loss) simultaneously. First, a normalization of the currents $Z_{i}^{n}\left(t\right)$ is able to control the spike number, with its details being given in Section 3.3.1. Second, the error term $\Delta_{i}^{n}\left(t\right)$ will accumulate in deeper layers, causing a lower spiking rate in the output layer (Rueckauer et al., 2017). The thresholding technique in Section 3.3.2 will be able to reduce the impact of such an error. Third, we need to maintain the consistency between CNN and SNN so that the above control can be effective, as in Section 3.3.3.

The input is encoded into a spike train *via* Poisson event-generation process (Sengupta et al., 2019) or interpreting the input as constant currents (Rueckauer et al., 2017). In this article, we select the latter.

### 3.3. ECC-Based Conversion Techniques

**Figure 2:**
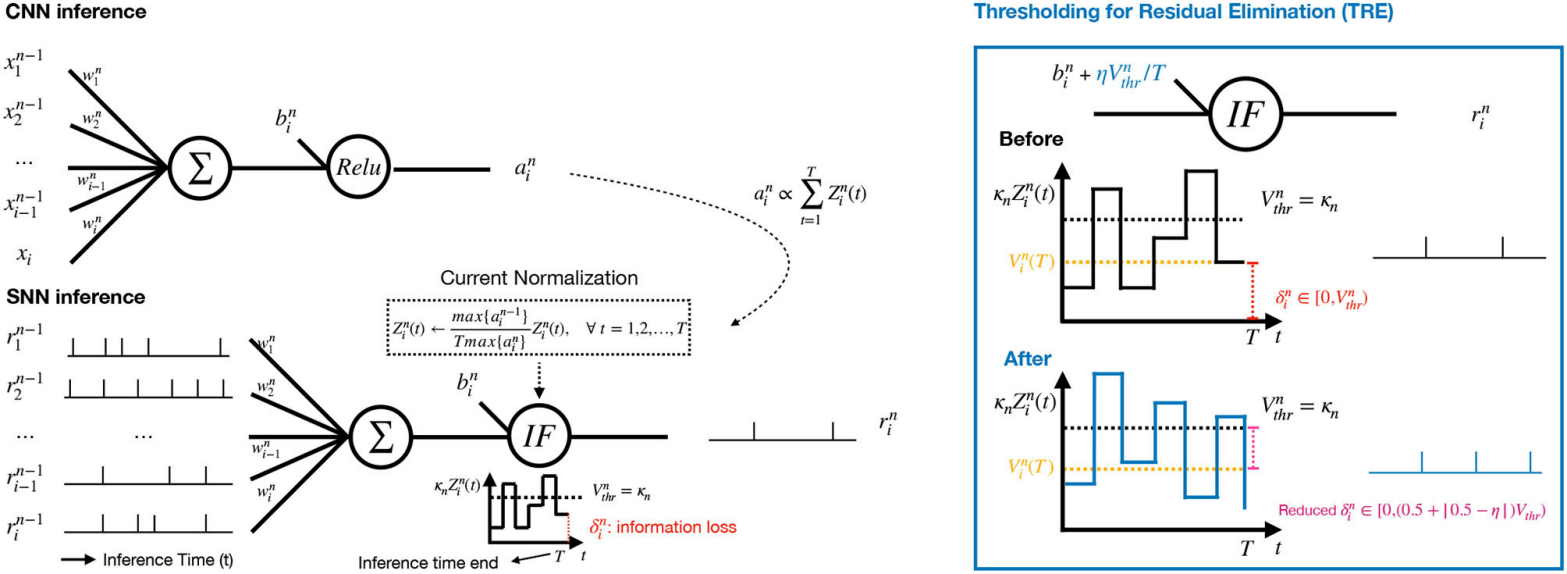
**Left**: Our proposed CNN-to-SNN conversion for the *n*-th layer with a current normalization component and a thresholding mechanism. The activation $a_{i}^{n}$ in the CNN (Top) is used for current normalization in the SNN (Bottom). **Right**: The proposed Thresholding for Residual Elimination (TRE) and the illustration of error reduction by TRE.

We develop three ECC-based techniques, including current normalization (CN), thresholding for residual elimination (TRE), and consistency maintenance for batch-normalization (CMB). Figure 2 illustrates CN and TRE, where the *n*-th layer of CNN is on the top and the corresponding conversed SNN layer is at the bottom. In the converted SNN layer, the sequences of spikes from the previous layer are aggregated, from which the current $Z_{i}^{n}\left(t\right)$ is accumulated in the neurons, and normalized by a factor (refer to Equation (7) below) to ensure that the increase of current at each timestep is within the range of [0,1]. The membrane potential $V_{i}^{n\left(t\right)}$ is produced according to Equation (2), followed by a spike generating operation as in Equation (4) once $V_{i}^{n}\left(t\right)$ exceeds the threshold $V_{thr}^{n}=\;\kappa_{n}$. The parameter κ_*n*_ is the current amplification factor, which will be explained in Section 3.3.1. The residual current $\Delta_{i}^{n}$ at the end of the spike train indicates the information loss in SNNs.

#### 3.3.1. Current Normalization

At layer *n*, before spike generation, CN normalizes the current $Z_{i}^{n}\left(t\right)$ by letting


(7) $$Z_{i}^{n}(t)\leftarrow \frac{\lambda_{n-1}}{T\lambda_{n}}Z_{i}^{n}(t),\;\forall \;t=1,\ldots ,T$$

where $\lambda_{n}≜\underset{i}{\max}\left\{a_{i}^{n}\right\}$ for *n* = 1, 2, …, *N*. We have λ_0_ = 1 when the input has been normalized into [0, 1] for every feature. The benefit of CN is 2-fold:

- By CN, the maximum number of spikes fed into the SNN is under control, i.e., we can have direct control of the energy consumption.
- It facilitates the use of a positive integer $V_{thr}^{n}=\kappa_{n}$ as the threshold to quantify the current, which is amplified by a factor of κ_*n*_, for spike generation. In doing so, the neuron with maximum current can generate a spike at every time step.

We randomly choose κ_*n*_ = 100 for $V_{thr}^{n}$ and normalize weights for all experiments, except quantised SNN. Since the scalar of quantized weights in each layer will be absorbed into the threshold, we will get a different threshold for each layer. The quantisation process is explained in Section 4.4.

To achieve CN, the following conversion can be implemented to normalize weights and bias as follows.


(8) $$W_{ij}^{n}\leftarrow \kappa_{n}\frac{\lambda_{n-1}}{\lambda_{n}}W_{ij}^{n},\;b_{i}^{n}\leftarrow \frac{\kappa_{n}b_{i}^{n}}{\lambda_{n}},\;\;V_{thr}^{n}\leftarrow \kappa_{n}.$$

Note that, the next layer will amplify the incoming current back to its original scale before its normalization. When κ_*n*_ = 1*or λ*_*n*_/λ_*n*−1_, the conversions correspond to weight normalization in Rueckauer et al. (2017) and threshold balancing in Sengupta et al. (2019), respectively.

#### 3.3.2. Thresholding for Residual Elimination

According to Equation (6), the error increment after conversion is mainly caused by the residual information, $\delta_{i}^{n}\left(T\right)\in \left[0,V_{thr}^{n}\right]$, which remains with each neuron after *T* timesteps and cannot be forwarded to higher layers. To mitigate such errors, we propose a technique TRE to keep $\delta_{i}^{n}\left(T\right)$ under a certain value (half of $V_{thr}^{n}$ as in our experiments). In particular, we add extra current to each neuron in order to have $\eta V_{thr}^{n}$ increment on each membrane potential, where η∈[0, 1). Specifically, we update the bias term $b_{i}^{n}$ of neuron *i* at layer *n* as follows


(9) $$\begin{array}{c} b_{i}^{n}(t):=b_{i}^{n}(t)+\eta V_{thr}^{n}/T \end{array}$$

for every timestep *t*. Intuitively, we slightly increase synaptic bias for every neuron at every step so that a small volume of current is pumped into the system continuously.

The following proposition says that this TRE technique will be able to achieve a reduction of error range, which directly lead to the improvement in the accuracy loss.

** Proposition 2**. Applying TRE will lead to


(10) $$\Theta_{i}^{n}(T)=\{\begin{array}{cc} 1, & \text{if}V_{i}^{n}(T)>(1-\eta )V_{thr}^{n}\\ 0, & \text{otherwise.} \end{array}$$

for timestep *T* as opposed to Equation (4). By achieving this, the possible range of errors is reduced from $\left[0,V_{thr}^{n}\right)$ to $\left[0,\left(0.5+|0.5-\eta |\right)V_{thr}^{n}\right)$.

We remark that, deploying TRE will increase at most one spike per neuron at the first layer and continue to affect the spiking rate at higher layers. This is the reason why we have slightly more spike operations than Rueckauer et al. (2017), as shown in Supplementary Figure 1C. A typical value η is 0.5.

#### 3.3.3. Consistency Maintenance for Batchnormalization

Batch normalization (BN) (Ioffe and Szegedy, 2015) accelerates the convergence of CNN training and improves the generalization performance. The role of BN is to normalize the output of its previous layer, which allows us to add the normalized information to weights and biases in the previous layer. We consider a conversion technique CMB to maintain the consistency between SNN and CNN in operating the BN layer, by requiring a constant for numerical stability ϵ, as follows.


(11) $$\begin{array}{c} \begin{array}{c} {\hat{W}}_{ij}^{n}=\frac{\gamma_{i}^{n}}{\sqrt{\sigma_{i}^{n2}+\epsilon }}W_{ij}^{n} \end{array} \end{array}$$

(12) $${\hat{b}}_{i}^{n}=\frac{\gamma_{i}^{n}}{\sqrt{\sigma_{i}^{n2}+\epsilon }}(b_{i}^{n}-\mu_{i}^{n})+\beta_{i}^{n}$$

where $\gamma_{i}^{n}$ and $\beta_{i}^{n}$ are two learned parameters, $\mu_{i}^{n}$ and $\sigma_{i}^{n}$ are mean and variance. ϵ is platform dependent: for Tensorflow, it is default as 0.001, and for PyTorch, it is 0.00001. The conversion method in Rueckauer et al. (2017) does not consider ϵ, and we found through several experiments that a certain amount of accuracy loss can be observed consistently. **Figure 5** shows the capability of CMB in reducing the accuracy loss.

## 4. Experiment

We implement the ECC method and conduct an extensive set of experiments to validate it. We consider its comparison with the state-of-the-art CNN-to-SNN conversion methods on images and DVS inputs (Sections 4.2, 4.5, respectively), the demonstration of its working with batch-normalization (Section 4.3), its robustness with respect to hardware deployment (Section 4.4), and an ablation study (Section 4.6). Due to the space limit, we present a subset of the results—the Supplementary Material includes more experimental results. We fix κ_*n*_ = 100 and ϵ = 0.001 throughout the experiments.

**Figure 3:**
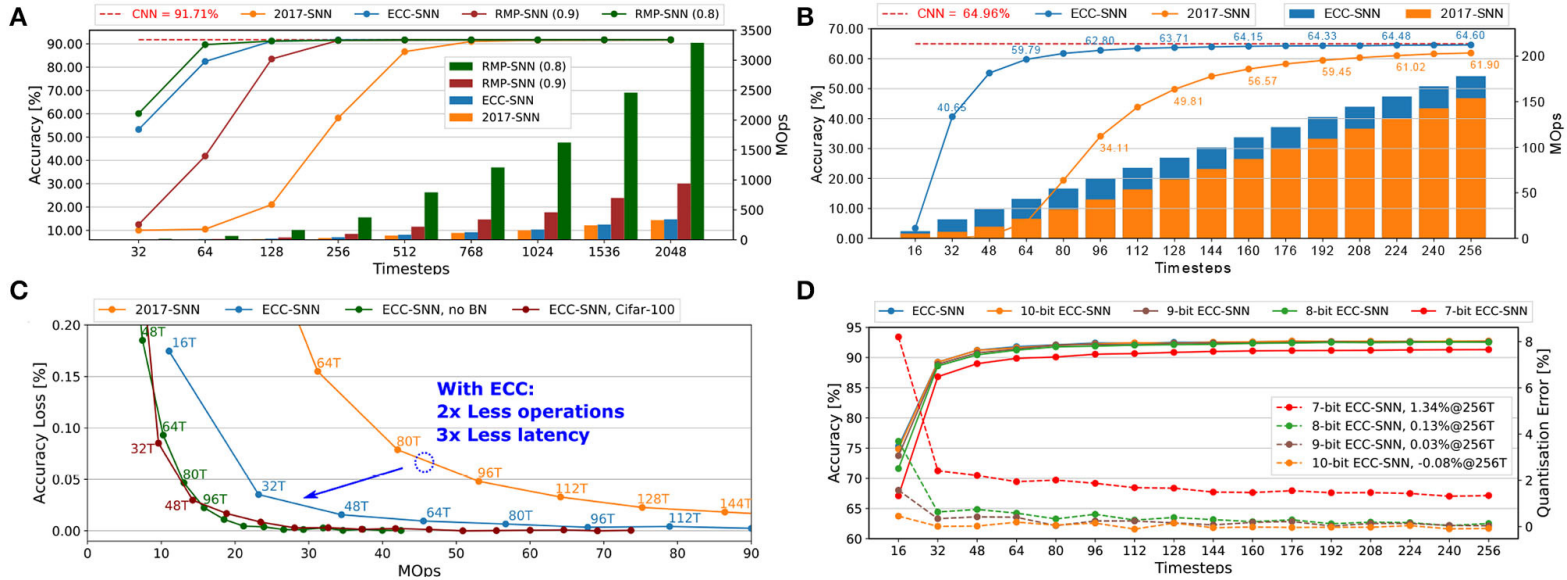
**(A)** Accuracy and energy consumption (MOps) with respect to timesteps for CIFAR-10. **(B)** Accuracy and energy consumption (MOps) with respect to timesteps, for ImageNet (Top-1 Acc). **(C)** Accuracy loss and latency with respect to energy consumption (MOps), for CIFAR-10 and CIFAR-100. **(D)** Accuracy and quantisation error with respect to timesteps, for CIFAR-10.

In this section, “2017-SNN” denotes the method proposed in Rueckauer et al. (2017). “RMP-SNN(0.8)” and “RMP-SNN(0.9)” denote the method in Han et al. (2020), with different parameters 0.8 or 0.9 as co-efficient to *V*_*thr*_. ‘ECC-SNN' is our method. We remark that it is shown in Han et al. (2020) that its conversion method outperforms that of Sengupta et al. (2019), so we only compare with Han et al. (2020). Moreover, we may write “Method@*n*T” to represent the specific ‘Method' when considering the spike trains of length *n*. Note that, only the CNN model in Figure 3A was trained without bias and BN, in order to have a fair comparison with RMP-SNN techniques. Since BN layers play an important role in training a high performance CNN and have the benefit of lowering the latency (c.f. Section 4.3), we believe it is essential to include them in CNN training. Therefore, we do not compare with Han et al. (2020) (i.e., RMP-SNN) and Sengupta et al. (2019) in other experiments because they do not work with BN.

Before proceeding, we explain how to estimate energy consumption. For CNNs, it is estimated through the multiply-accumulate (MAC) operations


(13) $$MAC\;operations\;for\;CNNs:\sum_{n=1}^{N}(2f_{in}^{n}+1)M^{n}$$

where $f_{in}^{n}$ is the number of input connections of the *n*-th layer. The number of MAC operations is fixed when the architecture of the network is determined. For SNNs, the synaptic operations are counted to estimate the energy consumption of SNNs (Merolla et al., 2014; Rueckauer et al., 2017) as follows.


(14) $$Synaptic\;operations\;for\;SNNs:\sum_{t=1}^{T}\sum_{n=1}^{N}f_{out}^{n}s^{n}$$

where $f_{out}^{n}$ is the number of output connections and *s*^*n*^ is the average number of spikes per neuron, of the *n*-th layer.

### 4.1. Experimental Settings

We work with both image datasets [CIFAR-10/100 (Krizhevsky and Hinton, 2009) and ImageNet (Russakovsky et al., 2015)] and DVS datasets (CIFAR-10-DVS Li et al., 2017) on several architectures (Simonyan and Zisserman, 2014) (VGG-16, VGG-19, and VGG-7). All the experiments are conducted on a CentOS Linux machine with two 2080Ti GPUs and 11 GB memory.

### 4.2. Comparisons With State-of-the-Art

Figure 3A presents a comparison between 2017-SNN, RMP-SNN, and ECC-SNN on both accuracy and energy consumption with respect to the timesteps, on VGG-16 and CIFAR-10. We note that both RMP-SNN and ECC-SNN outperform 2017-SNN, in terms of the number of timesteps to reach near-zero accuracy loss. Furthermore, ECC-SNN is better than RMP-SNN(0.9) and competitive with RMP-SNN(0.8) in terms of reaching near-zero accuracy loss under certain latency. Specifically, both ECC-SNN and RMP-SNN(0.8) require 128 timesteps and RMP-SNN(0.9) requires 256 timesteps. Importantly, we note that both RMP-SNN(0.8) and RMP-SNN(0.9) consume much more energy, measured with MOps, than ECC-SNN. Actually, ECC-SNN does not consume significantly more energy than 2017-SNN.

Similar results can be extended to a large dataset such as ImageNet. Moreover, to investigate further into the energy consumption, Figure 3B presents a comparison with 2017-SNN. All the above results show that ECC-SNN significantly reduces the latency, easily reaches the near-zero loss, and costs a minor increase in energy.

The above results, together with those in Supplementary Figures 1A,C,D,E), reflect exactly the advantage of using ECC-SNN. That is RMP-SNN(0.8) and ECC-SNN are the best in achieving near-zero accuracy loss with low latency, but RMP-SNN requires significantly more energy than the other two methods. Therefore, *ECC-SNN achieves the best when considering energy, latency, and accuracy loss*.

Batch-normalization (BN) has become indispensable to train CNNs, so we believe a CNN-to-SNN method should be able to work with it. After demonstrating a clear advantage over RMP-SNN, for the rest of this section, we will focus on the comparison with 2017-SNN, which deals with BN. We trained CNNs using Tensorflow by having a batch-normalization layer after each convolutional layer.

### 4.3. Batch-Normalization

Figure 3C considers the impact of working with BN. Compared with 2017-SNN, ECC-SNN achieves similar accuracy loss by taking 2x less MOps and 3x less latency. Moreover, to achieve the same accuracy loss, ECC-SNN without BN, i.e., ECC applies on CNNs without BN layers, requires significantly more timesteps, with slightly less MOps. Moreover, our other experiments show that RMP-SNN (0.8), without BN in its method, can only achieve 48.32% in 256T. With BN, 2017-SNN can achieve 49.81% in 128T. ECC-SNN further improves on this, achieving 63.71% in 128T. That is, *batch-normalization under ECC-SNN can help reduce the latency*. This is somewhat surprising, and we believe further research is needed to investigate the formal link between BN and latency.

### 4.4. Robustness to Quantisation

Figure 3D and Supplementary Figure 1B present how the change in the number of bits to represent weights may affect the accuracy and the quantisation error. This is an important issue, as the SNNs will be deployed on the neuromorphic chip, such as Loihi (Davies et al., 2018) and TrueNorth (Akopyan et al., 2015), or FPGA, which may have different configurations. For example, Loihi can have weight precision at 1-9 bits. Floating-point data, both weights and threshold can be simply converted into fixed-point data after CN in two steps: normalizing the weights into the range [-1,1] and scaling the threshold using the same normalization factor, and then multiplied with 2^*b*^, where *b* is the bit width (Ju et al., 2019; Sze et al., 2019). From Figure 3D and Supplementary Figure 1B, the reduction from 32-bit to 10-, 9-, 8-, and 7-bit signed weights does lead to a drop in the accuracy, but unless it goes to 7-bit, the accuracy loss is negligible. This shows that *our ECC method is robust to hardware deployments*.

### 4.5. DVS Dataset

**Figure 4:**
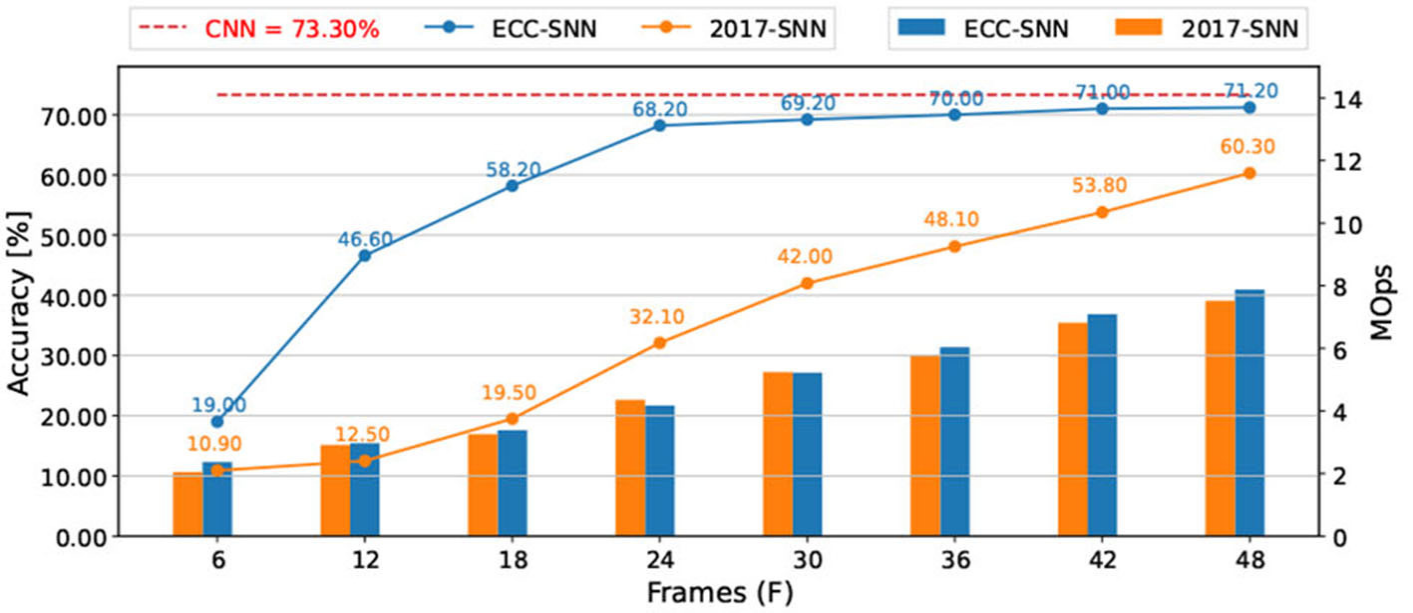
Accuracy and energy consumption (MOps) with respect to frames, between 2017-SNN and ECC-SNN, for CIFAR-10-DVS, and VGG-7.

CIFAR-10-DVS (Li et al., 2017) is a benchmark dataset of DVS inputs, consisting of 10,000 inputs extracted from the CIFAR-10 dataset using a DVS128 sensor. The resolution of data is 128x128. We preprocess the data following Wu et al. (2021) and Kugele et al. (2020), select the first 1.3 s of the event stream and down-scale the input into 42 x 42. For each dimension of input, we calculate the number of spikes over the 1.3 s simulation and normalize with a constant representing the maximum number of spikes. During SNN processing, as shown in Figure 1B, the latency is based on spikes. The experiments using VGG7 (Figure 4), VGG16, and ResNet-18 (Sengupta et al., 2019; Supplementary Figures 2A,B) show that ECC-SNN performs much better than 2017-SNN, who also work with BN layer. Moreover, in Table 2, we compare few methods including a recent direct training method and highlight the best results for each objective. We can see that ECC-SNN can always achieve better accuracy, less frames, and less energy consumption.

**Table 2:** Comparison of SNN accuracy, latency, and energy consumption (MOps), between direct training, 2017-SNN and ECC-SNN, for Cifar-10-DVS.

**Method**	**Publication**	**Accuracy**	N${}_{f}^{*}$	**MOps**
Direct training (VGG7)	Wu et al., 2021	62.50	-	-
2017-SNN (DenseNet)	Kugele et al., 2020	65.61	60	1,551
ECC-SNN (VGG16)	this paper	71.20	**48**	66.79
ECC-SNN (VGG7)	this paper	**71.30**	**48**	**7.52**

Moreover, we recall the results shown in Figure 1 concerning the comparison between DVS inputs and images. For the same problem, if we choose the deployment workflow of “training a CNN → converting into an SNN → deploying on edge devices with e.g., event camera,” we may consume 10+ times less energy (7.52 vs. 90 MOps for CIFAR-10) by taking DVS inputs. Both are in stark contrast with the other deployment workflow “training a CNN → deploying on edge devices with camera,” which costs much more energy (307MOPs and 657MOps, respectively).

### 4.6. Ablation Study

**Figure 5:**
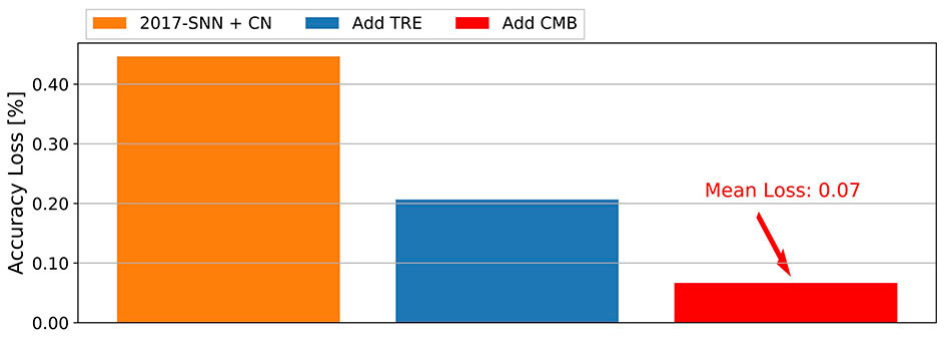
Contribution of CN, CMB, and TRE to the reduction of mean accuracy loss, for CIFAR-10 and VGG-16.

To understand the contributions of the three ingredients of ECC-SNN, i.e., CN, CMB, and TRE, we conduct an experiment on VGG-16 and CIFAR-10, by gradually including technical ingredients to see their respective impact on the accuracy loss. Figure 5 shows the histograms of the mean accuracy losses in 256T, over the 281–283th epochs. We see that every ingredient plays a role in reducing the accuracy loss, with the TRE and CN being lightly Moreover, We also consider the impact of η (as in Supplementary Figure 1F).

## 5. Conversion Optimized Through Distribution-Aware CNN Training

Up to now, all methods we discussed and compared, including our ECC method, are focused on optimizing the CNN-to-SNN conversion, without considering whether or not the CNN itself may also play a role in eventually obtaining a good-performing SNN. In this section, we will discuss several recent techniques that include the consideration of CNN training and show that our ECC method can also improve them by optimizing the CNN-to-SNN conversion.

As noted in Section 3.3.1 that the maximum value of activations is a key parameter in the conversion. Based on this, Rueckauer et al. (2017) and Lu and Sengupta (2020) suggest that a particular percentile from the histogram on the CNN activation may improve conversion efficiency. One step further, Yu et al. (2020) suggest that a good distribution with less outliers on CNN activation can be useful for quantisation. Therefore, we call these techniques distribution-aware CNN training techniques, to emphasise that they are mainly focused on optimizing the CNN training through enforcing good distributions on the activations.

**Figure 6:**
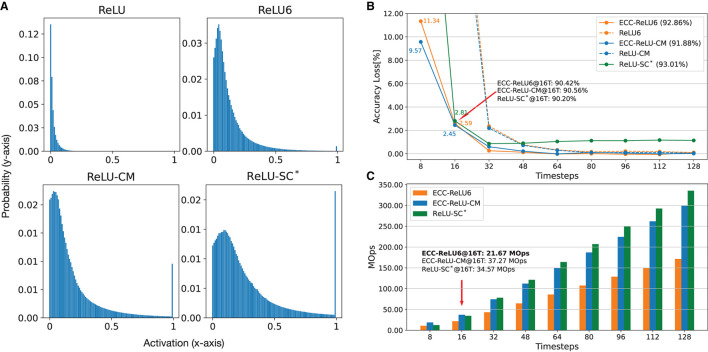
Comparison of SNNs using different clipping methods, ReLU6 (Jacob et al., 2018; Lin et al., 2019), ReLU-CM (Yu et al., 2020), ReLU-SC^*^ (Deng and Gu, 2021), for CIFAR-10 on VGG-16. **(A)** Normalized activation distribution of the first layer. **(B)** Accuracy with respect to timesteps. **(C)** Energy consumption (MOps) with respect to timesteps. ^*^We use ReLU-SC to train an SNN with a fixed timestep (16T), as it does not need extra training.

To show that our ECC method is complementary to the distribution-aware CNN training techniques, we implement some existing CNN training techniques that can affect activation distribution and show that ECC can also work with them to achieve optimized conversion. Specifically, Yu et al. (2020) notice that the clipped ReLU can enforce small activation values (i.e., close to zero) to become greater, and eventually reshape the distribution from a Gaussian-like distribution to a uniform distribution. We follow this observation to train CNN models with different clipping methods, including ReLU6 (clipped by 6) from Lin et al. (2019) and Jacob et al. (2018), ReLU-CM (clipped by k-mean) from Yu et al. (2020), and ReLU-SC (shift and clipped) from Deng and Gu (2021). Figure 6A shows that all clipping methods can shift the original activation value (as in the top left figure) to values closer to the mean value (i.e., near the peak area). Such a shift of maximum activation value can significantly reduce the possibility for the maximum value to become an outlier.

Figure 6B presents a comparison between SNNs obtained through ReLU6, ReLU-CM and ReLU-SC, with and without the application of ECC. First of all, distribution-aware training can improve performance. For example, with clipping methods, the accuracy loss is less than 2.2%@32T, which is better than ECC-SNN (3.2%@32T) as in Supplementary Figure 1C. Then, we can see that, with ECC, ReLU6 and ReLU-CM can achieve 2x less latency with little performance degradation. Although ECC-ReLU6, ECC-ReLU-CM, and ReLU-SC achieve similar accuracy (90.20–90.56%@16T), ECC-ReLU-CM has the best adaptability to different timesteps. By contrast, ECC-ReLU6 uses 1.5–1.7x less operations at 16T than ReLU-SC and ECC-ReLU-CM, as shown in Figure 6C. We only apply ECC to ReLU6 and ReLU-CM, as ReLU-SC in Deng and Gu (2021) is not designed to be adaptable to different timesteps.

The above results show that distribution-aware CNN training and our ECC method can both improve the CNN-to-SNN conversion. While distribution-aware CNN training can reduce the accuracy loss, the application of the ECC method can further improve the performance of the resulting SNN model. Furthermore, it is worth mentioning that ECC can take the advantage of the accumulated bias current to optimize a single SNN model with respect to different timesteps.

## 6. Discussion

###  Variants to the Unifying Framework

The current unifying framework (Section 3.2) considers the ReLU activation function, which exhibits a linear relation between accumulated current and spiking rate. There are other—arguably more natural—features in biological neurons, such as a leak, refractory time, and adaptive threshold, as discussed in Kobayashi et al. (2009). If considering these features, the relation between accumulated current and spiking rate will become non-linear. To deal with them, it can be an interesting future work to consider extending the unifying framework to address the connection between nonlinear activation functions (e.g., sigmoid) on CNN and the dynamic properties on SNN.

### Hyper-Parameters in ECC

Most of the hyper-parameters in ECC-SNN are determined with reasons, such as κ_*n*_ (Section 3.3.1) and η (Section 3.3.2), while timesteps (T) are determined by practical application according to e.g., required accuracy. Although some gradient-based optimization methods, such as Rathi et al. (2020), Rathi and Roy (2021), and Li et al. (2021), can improve the SNN to a fixed timestep, ECC allows SNN to be adaptive to different timesteps. In the future, we will consider hyper-parameters tuning methods, e.g., Parsa et al. (2020), to further improve ECC-SNN while maintaining its adaptability.

## 7. Conclusion

We develop a unifying theoretical framework to analyze the conversion from CNNs to SNNs and a new conversion method ECC to explicitly control the currents, so as to optimize accuracy loss, energy efficiency, and latency simultaneously. By comparing state-of-the-art methods, we confirm the superior performance of our method. Moreover, we study the impact of batch-normalization and show the robustness of ECC over quantization.

## Data Availability Statement

The original contributions presented in the study are included in the article/Supplementary Material, further inquiries can be directed to the corresponding author.

## Author Contributions

DW designed the study, contributed to the source code, conducted the experiments, and evaluated the results. XY and XH provided the feedback and scientific advice throughout the process. All authors contributed to the final manuscript.

## Funding

DW is supported by the University of Liverpool and China Scholarship Council Awards (Grant No. 201908320488). This project has received funding from the European Union's Horizon 2020 Research and Innovation Programme under Grant Agreement No. 956123. It is also supported by the U.K. EPSRC (through End-to-End Conceptual Guarding of Neural Architectures [EP/T026995/1]). 
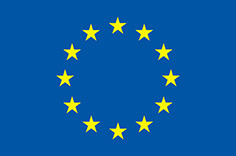


## Conflict of Interest

The authors declare that the research was conducted in the absence of any commercial or financial relationships that could be construed as a potential conflict of interest.

## Publisher's Note

All claims expressed in this article are solely those of the authors and do not necessarily represent those of their affiliated organizations, or those of the publisher, the editors and the reviewers. Any product that may be evaluated in this article, or claim that may be made by its manufacturer, is not guaranteed or endorsed by the publisher.

## Footnotes

^1^ https://github.com/Dengyu-Wu/spkeras

^2^The activation values in the original CNNs can be represented by the current through the analog/digital circuits in the resulting SNNs, so that controlling current through spike train in SNNs corresponds to data flow operations in CNNs.
