# Supplementary material for: A Little Energy Goes a Long Way: Build an Energy-Efficient, Accurate Spiking Neural Network From Convolutional Neural Network
Source: Front Neurosci. 2022 May 26;16:759900. doi: 10.3389/fnins.2022.759900 (PMC9179229; doi:10.3389/fnins.2022.759900)
Supplement: Supplementary file 1 [file Data_Sheet_1.PDF]

Supplementary Material

The supplementary material of this submission includes this document and a software package SpKeras. This document contains technical proofs for Propisiton 1 and Proposition 2, as well as some additional experiments. The software package includes source codes and its documentation (software framework, README, etc), as well as an example for VGG16 network on CIFAR-10 dataset.

1 PROOF OF PROPOSITION 1

According to Equation (2), the accumulated current of i-th neuron at layer n over the simulation time T can be calculated as

$$\sum_{t=1}^{T} V_i^n(t) = \sum_{t=1}^{T} (V_i^n(t-1) + Z_i^n(t) - \Theta_i^n(t) V_{thr}^n). \tag{S1}$$

By using $N_i^n(T) = \sum_{t=1}^{T} \Theta_i^n(t)$, we obtain

$$N_i^n(T) V_{thr}^n = \sum_{t=1}^{T} Z_i^n(t) - (\sum_{t=1}^{T} V_i^n(t) - \sum_{t=1}^{T} V_i^n(t-1)) \tag{S2-a}$$

which further yields

$$\frac{N_i^n(T)}{T} = \frac{1}{T V_{thr}^n} \sum_{t=1}^{T} Z_i^n(t) - \frac{1}{T V_{thr}^n} (V_i^n(T) - V_i^n(0)). \tag{S2-b}$$

According to Equation (3), the current accumulated in hidden layers can be calculated as

$$\sum_{t=1}^{T} Z_i^n(t) = \sum_{j=1}^{M^{n-1}} W_{ij}^n N_j^{n-1}(T) + T b_i^n. \tag{S3}$$

By inserting Equation (S3) into Equation (S2-b) $(n > 1)$ and setting $V(0) = 0$, the spiking rate, $r_i^n(T) = N_i^n(T)/T$, can be iteratively computed by

$$r_i^n(T) = \frac{1}{V_{thr}^n} \Big(\sum_{j=1}^{M^{n-1}} W_{ij}^n r_j^{n-1}(T) + b_i^n \Big) - \frac{V_i^n(T)}{T V_{thr}^n}. \tag{S4}$$

Given the condition in Equation (5), the iterative spiking rate computation can be initialised at first layer $(n = 1)$ as

$$r_i^1(T) = \frac{a_i^1}{V_{thr}^1} - \frac{V_i^1(T)}{T V_{thr}^1} \tag{S5}$$

In general, for any $t \in [1, \ldots, T]$, the accumulated spiking rate at t can be induced as

$$r_i^n(t) = \frac{1}{V_{thr}^n} \Big(\sum_{j=1}^{M^{n-1}} W_{ij}^n r_j^{n-1}(t) + b_i^n \Big) - \frac{V_i^n(t)}{t V_{thr}^n} \tag{S6}$$

where the spiking rate of the first layer can be initialised as

$$r_i^1(t) = \frac{a_i^1}{V_{thr}^1} - \frac{V(t)}{tV_{thr}^1}. \tag{S7}$$

This completes the proof of Proposition 1.

2 PROOF OF PROPOSITION 2

Adding the updated bias term of Equation (9) into Equation (S4), we have

$$\begin{aligned} r_i^n(T) = \frac{1}{V_{thr}^n}\Big(\sum_{j=1}^{M^{n-1}} W_{ij}^n r_j^{n-1}(T) + b_i^n\Big) \\ - \frac{V_i^n(T) - (1-\eta)V_{thr}^n}{TV_{thr}^n} \end{aligned} \tag{S8}$$

As such, the residual spiking rate due to the residual current turns out to be

$$\Delta_i^n(T) = \frac{V_i^n(T) - (1-\eta)V_{thr}^n}{TV_{thr}^n}. \tag{S9}$$

If $\Delta_i^n(T) \geq 0$, an extra spike can be generated. This yields the thresholding rule in Proposition 2.

Moreover, we note that, with thresholding and η, the residual current will be less than $(0.5+|0.5-\eta|)V_{thr}^n$. Actually, any neuron with more than $(0.5+|0.5-\eta|)V_{thr}^n$ current will automatically incur a spike and lead to a reduced residual current within $[0, (0.5+|0.5-\eta|)V_{thr}^n)$. Together with the previous argument for thresholding rule, this completes the proof of Proposition 2.

Intuitively, Proposition 2 can be explained as follows. The extra current is injected into the SNN system – in the form of increased bias term – to raise the level of residual current beyond the predetermined threshold V_{thr}^n. Both the increased residual current and the η will take effect in increasing the possibility of generating one extra spike. This is equivalent to decrease the effective threshold at the final timestep T for the residual current, as in Equation (10). This process ensures that the error caused by the residual current can be reduced, because technically the range of residual current retained in the neurons is reduced from $[0, V_{thr}^n)$ to $[0, 0.5+|0.5-\eta|V_{thr}^n)$.

3 MORE EXPERIMENTAL RESULTS COMPARING TO STATE-OF-THE-ART

Figure S1A shows the accuracy impact of small timesteps, for both ECC-SNN and 2017-SNN. We plot the accuracy of 10 epochs when the CNN training is almost converged, i.e., when the accuracy does not fluctuate significantly with the advance of epochs. For 2017-SNN, a smaller timestep may lead to significant performance drop. For example, the accuracy of 2017-SNN@256T is around 92.5%, while 2017-SNN@128T is around 91.5%, a 1% accuracy gap. On the other hand, ECC-SNN does not experience significant accuracy drop. *This observation supports our view that we may not need a significant amount of energy (here, the timesteps) to reach an acceptable level of accuracy loss.*

The last point is further exhibited in Figure S1C, where we compare only ECC-SNN with 2017-SNN. We can see that, only a minor energy consumption increase from 2017-SNN to ECC-SNN, but ECC-SNN can achieve near-zero accuracy loss with much smaller timesteps.

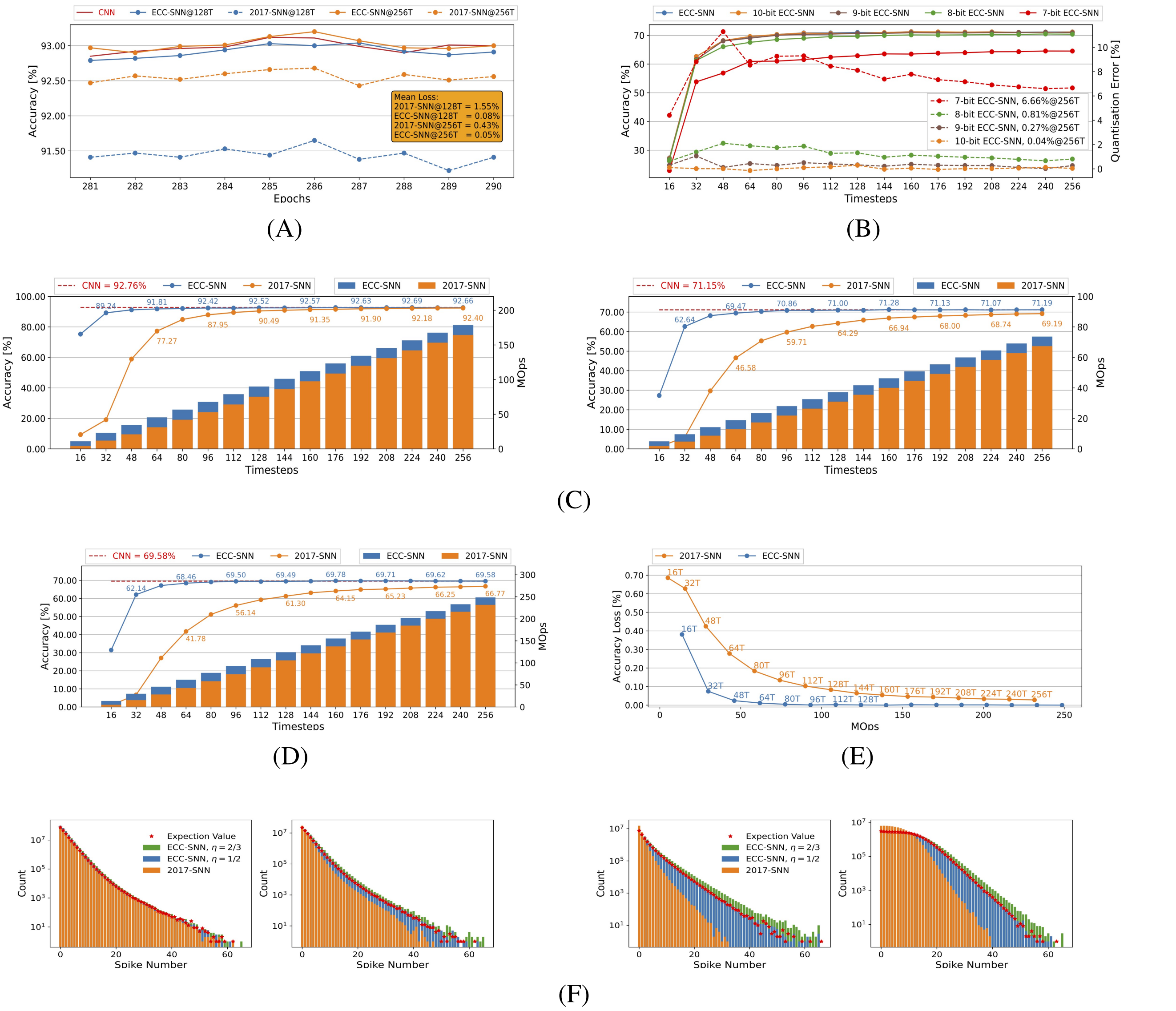

Figure S1: Results for Cifar-10/100, **(A)**Accuracy w.r.t. training epochs, on SNNs (2017-SNN vs ECC-SNN), for CIFAR-10 (average loss in yellow box). **(B)** Accuracy and quantisation error w.r.t. timesteps, for CIFAR-100. **(C)** VGG-16: Accuracy and energy consumption (MOps) w.r.t. timesteps, between 2017-SNN and ECC-SNN, for CIFAR-10 (left) and CIFAR-100 (right). **(D)** Accuracy and energy consumption (MOps) w.r.t. timesteps, for CIFAR-100 on VGG-19. **(E)** Accuracy loss and latency w.r.t. energy consumption (MOps), for CIFAR-100 on VGG-19. **(F)** Spike count on 4th, 8th, 12th and 16th layer (from left to right) with 64 timesteps for CIFAR-100.

4 EXPERIMENT ON VGG-19 ARCHITECTURE

The following experiment on CIFAR-100 and VGG-19 shows that 80 timesteps has been sufficient for ECC-SNN to achieve near-zero accuracy loss. On the other hand, since VGG-19 is deeper than VGG-16, the accuracy loss of 2017-SNN Rueckauer et al. (2017) becomes more significant (See Figure S1D).

Moreover, we note that, comparing to 2017-SNN, ECC-SNN only has a very minor energy consumption increase, but ECC-SNN can achieve near-zero accuracy loss with much smaller timesteps (See Figure S1E).

That is, ECC-SNN can achieve similar accuracy performance by using much less spike operations and shorter latency. *These observations confirm the results we have for VGG-16 architecture*.

5 EXPERIMENT ON DVS-CIFAR-10

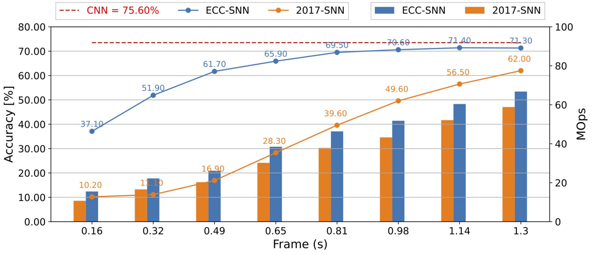

(A)

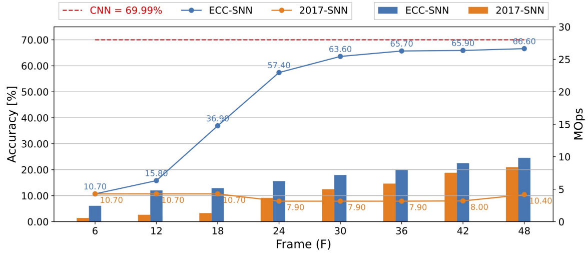

(B)

Figure S2: Accuracy and energy consumption (MOps) w.r.t. frames, between 2017-SNN and ECC-SNN, for CIFAR-10-DVS on VGG-16 (A) and ResNet-18 (B).

Figure S2B indicates that ECC-SNN is also applicable to other network architectures, such as ResNet-18. The conversion of ResNet introduced in Sengupta et al. (2019) requires the same normalisation factor for those layers connected to the same ReLU junction. The ReLU junction sums its input layers to create residual connection. To control the latency in hidden layers, the maximum value of the junction determines the normalisation factor of its input layers. Even though the converted ResNet-18 may be affected by the outliers in CNN activation (e.g. both low accuracy and MOps for 2017-SNN), we can still observe the improvement of using ECC-SNN.

6 HOW η AFFECTS THE NUMBER OF SPIKES

Figure S1F presents the spike count of hidden layers, for 2017-SNN and ECC-SNN with two different η, for CIFAR-100. We note that, on the shadow layers, the spike count increase of ECC-SNN over 2017-SNN is minor. It becomes more significant for deeper layers. The increased spikes help ECC-SNN to follow the expecting distribution more closely. The expectation curve is extracted by encoding CNNs activation in each layer directly, which is without impact of Δ_i^n and represents the optimal case. When increasing η from 1/2 to 2/3, the spike count distribution deviates further from its expectation.

REFERENCES

Rueckauer, B., Lungu, I., Hu, M., Y.and Pfeiffer, and Liu, S. (2017). Conversion of continuous-valued deep networks to efficient event-driven networks for image classification. *Frontiers in neuroscience* 11, 682

Sengupta, A., Ye, Y., Wang, R., Liu, C., and Roy, K. (2019). Going deeper in spiking neural networks: Vgg and residual architectures. *Frontiers in neuroscience* 13, 95
